# Supplementary material for: A Comprehensive Assessment of Ultraviolet-Radiation-Induced Mutations in Flammulina filiformis Using Whole-Genome Resequencing
Source: J Fungi (Basel). 2024 Mar 20;10(3):228. doi: 10.3390/jof10030228 (PMC10971301; doi:10.3390/jof10030228)
Supplement: Supplementary file 1 [file jof-10-00228-s001.zip › Supplementary Material S8/KEGG annotation/out/64381550635650.os/KO/out_map/map04713.html]

KEGG PATHWAY: Circadian entrainment - Reference pathway


|  |  |
| --- | --- |
| **Circadian entrainment - Reference pathway** |  |

[
Pathway menu
| Organism menu
| Pathway entry
| Show description
| User data mapping
]

Circadian entrainment is a fundamental property by which the period of the internal biological clock is entrained by recurring exogenous signals, such that the organism's endocrine and behavioral rhythms are synchronized to environmental cues. In mammals, a master clock is located in the suprachiasmatic nuclei (SCN) of the hypothalamus and may synchronize circadian oscillators in peripheral tissues. Light signal is the dominant synchronizer for master SCN clock. Downstream from the retina, glutamate and PACAP are released and trigger the activation of signal transduction cascades, including CamKII and nNOS activity, cAMP- and cGMP-dependent protein kinases, and mitogen-activated protein kinase (MAPK). Of non-photic entrainment, important phase shifting capabilities have been found for melatonin, which inhibits light-induced phase shifts through inhibition of adenylate cyclase (AC). Multiple entrainment pathways converge into CREB regulation. In turn, phosphorylated CREB activates clock gene expression.

|  |  |
| --- | --- |
| Reference pathway | 100% |
